# Differences in molecular evolutionary rates among microRNAs in the human and chimpanzee genomes

**DOI:** 10.1186/s12864-016-2863-3

**Published:** 2016-07-29

**Authors:** Gabriel Santpere, Maria Lopez-Valenzuela, Natalia Petit-Marty, Arcadi Navarro, Yolanda Espinosa-Parrilla

**Affiliations:** 1 Department of Experimental and Health Sciences, IBE, Institute of Evolutionary Biology, (Universitat Pompeu Fabra -CSIC), Barcelona, Catalonia Spain; 2 Department of Neurobiology, Yale School of Medicine, New Haven, Connecticut USA; 3 Centre for Genomic Regulation (CRG), Barcelona, Catalonia Spain; 4 National Institute for Bioinformatics (INB), Barcelona, Catalonia Spain; 5 Institució Catalana de Recerca i Estudis Avançats (ICREA), Barcelona, Catalonia Spain; 6 School of Medicine, Universidad de Magallanes, Punta Arenas, Chile

**Keywords:** microRNAs, Acceleration rates, Primates, Divergence, microRNA cluster, Evolution

## Abstract

**Background:**

The rise of the primate lineage is accompanied by an outstanding emergence of microRNAs, small non-coding RNAs with a prominent role in gene regulation. In spite of their biological importance little is known about the way in which natural selection has influenced microRNAs in the human lineage. To study the recent evolutionary history of human microRNAs and to analyze the signatures of natural selection in genomic regions harbouring microRNAs we have investigated the nucleotide substitution rates of 1,872 human microRNAs in the human and chimpanzee lineages.

**Results:**

We produced a depurated set of microRNA alignments of human, chimpanzee and orang-utan orthologs combining BLAT and liftOver and selected 1,214 microRNA precursors presenting optimal secondary structures. We classified microRNAs in categories depending on their genomic organization, duplication status and conservation along evolution. We compared substitution rates of the aligned microRNAs between human and chimpanzee using Tajima’s Relative Rate Test taking orang-utan as out-group and found several microRNAs with particularly high substitution rates in either the human or chimpanzee branches. We fitted different models of natural selection on these orthologous microRNA alignments and compared them using a likelihood ratio test that uses ancestral repeats and microRNA flanking regions as neutral sequences. We found that although a large fraction of human microRNAs is highly conserved among the three species studied, significant differences in rates of molecular evolution exist among microRNA categories. Particularly, primate-specific microRNAs, which are enriched in isolated and single copy microRNAs, more than doubled substitution rates of those belonging to older, non primate-specific microRNA families.

**Conclusions:**

Our results corroborate the remarkable conservation of microRNAs, a proxy of their functional relevance, and indicate that a subset of human microRNAs undergo nucleotide substitutions at higher rates, which may be suggestive of the action of positive selection.

**Electronic supplementary material:**

The online version of this article (doi:10.1186/s12864-016-2863-3) contains supplementary material, which is available to authorized users.

## Background

During the last decade microRNAs (miRNAs), a class of small non-coding RNAs, have gained recognition as key posttranscriptional regulators of gene expression. These small RNAs are implicated in almost every biological process and their deregulation is associated with a large number of diseases, particularly with cancer [1, 2]. miRNA-mediated regulatory networks are large and complex given that a single miRNA can recognize numerous different messenger RNAs and, in turn, messenger RNAs tend to harbour target sites for multiple miRNAs [3, 4]. Thus, by directly or indirectly tuning the expression of their target genes, miRNA regulation can greatly influence cellular transcriptomes.

MiRNA genes are transcribed by RNA polymerase II as primary transcripts that are cleaved by the Drosha–DGCR8 complex into approximately 80 nucleotide (nt) miRNA precursors. These molecules fold into a hairpin structure that is exported to the cytoplasm where they are cleaved by the endoribonuclease Dicer to yield two separated pieces of single stranded RNAs of approximately 22 nt long. These are the actual functional miRNAs, called mature miRNAs -3p and -5p. Mature miRNAs coupled with the RNA-Induced Silencing Complex (RISC) lead the repression of their target genes either by obstruction of translation or by destabilization of messenger RNAs that are recognized in a sequence specific manner [5].

Novel miRNA genes appear in the genomes with relative ease as they only require of a transcription start site and a short sequence capable of forming a simple secondary structure that can be recognized by Drosha and Dicer. The genomic sources from which a miRNA gene can be generated are numerous [6, 7] and thousands of hairpins with miRNA-like properties are predicted to exist in the human genome [8]. Gene duplication, either in tandem or non-local, is a major source of novel miRNAs [9]. This phenomenon gives rise to numerous paralogous miRNAs that are grouped into miRNA families according to their sequence similarity [10]. As it happens with protein-coding genes, independent mutations in paralogous miRNA copies can lead to neofunctionalization and thus to the birth of a novel miRNA [11]. An interesting consequence of tandem duplication is the formation of miRNA clusters that are transcribed in a polycistronic fashion [12–14]. Around half of human miRNA clusters are composed of miRNAs belonging to the same miRNA family but there are also multi-family clusters, which are probably originated by *de novo* formation of hairpins on existent miRNA transcripts [14]. Most human miRNA clusters present from two to eight miRNAs with the notable exception of a cluster containing 42 miRNAs on chromosome 14 and another one on chromosome 19 holding 46 miRNAs [15]. Additionally, introns are another important source for new miRNAs that may be created from hairpins of intronic RNA, which are conveniently provided with the promoter of the host gene [16, 17]. Generation of miRNAs derived from transposable elements has also been reported in both plant and animals and, compared with miRNAs of other origin, miRNAs originated from transposable elements tend to be younger and lineage-specific [18, 19].

MiRNAs accumulate through time, being continuously added to metazoan genomes and rarely lost once fixed, which gives rise to lineage-specific miRNAs [9, 17, 20]. Other authors have shown that half of the newly born mammalian miRNA families would be lost by purifying selection along evolution [21]. The rate of miRNA acquisition has not been uniform over the time and includes additions and losses in an overall positive balance. Moreover, miRNA expansions are detected at evolutionary times that coincide with dramatic changes in body plans, the emergence of specialized tissues and other major phenotypic changes and divergences [22, 23]. Relevant miRNA expansions have been observed at the split of bilaterians [24], at the time of emergence of vertebrates [22] and the advent of eutherians [9].

The rise of primates is also marked by an outstanding expansion of miRNAs and, thus, a large fraction of primate miRNAs is specific of this lineage [25]. In fact, from all human miRNAs annotated so far only a small fraction (~15 %) is conserved beyond placental mammals. Most of human miRNAs originated at two evolutionary time points: the early phase of the radiation of placental mammals and the rise of the hominoid lineage [26]. The study of primate miRNAs has aimed mostly at the characterization of the miRNAs present in these species, first at the sequence level, with studies based on computational approaches [27, 28]; and more recently at the expression level by RNA sequencing of different tissues in various species [21, 29].

Given the clinical relevance and relative easiness to obtain samples, humans are by far the most deeply explored species. The current version of miRBase, the main repository for miRNAs [30], counts 1,881 miRNA precursors in human. This is roughly three times the number of miRNAs reported in other primates (*Pan troglodytes*, 655; *Gorilla gorilla*, 352; *Pongo pygmaeus*, 642; *Macaca mulatta*, 619) [miRBase 21, June 2014]. Additionally, two recent studies have found numerous novel human miRNAs (2,469 by Friedlander [31] and 3,500 by Londin [32]) not yet included in MiRBase but that increase the gap between human and non-human primates’ miRNAs.

Even though miRNAs are crucial regulatory elements for animal divergence and evolution, how natural selection has acted upon them has been poorly explored. Several studies show that human miRNAs are constrained and evolve under purifying selection [33, 34]. No positive selection has been demonstrated so far in miRNA regions, with the single exception of a region on human chromosome 14 that harbours numerous miRNAs and small nucleolar RNAs (snoRNAs) and shows signals of recent local positive selection [34]. In an attempt to analyze the signatures of natural selection on human miRNAs, we investigated the substitution rates of 1,872 human miRNAs in both human and chimpanzee genomes. We observed that, beyond the general constriction existing on miRNA sequences, primate-specific miRNAs, which tend to be isolated and single copy miRNAs, present higher evolutionary substitution rates than older miRNAs, which have a tendency to be arranged in clusters. We suggest that miRNA-driven evolution in primates can be in part sustained by mutation in novel, primate-specific miRNAs, which may reflect the action of positive selection. These results help to identify miRNA attributes that have been specially constrained, relaxed or accelerated along primate evolution.

## Results and discussion

### Obtaining alignments of orthologous miRNAs

**Fig. 1:**
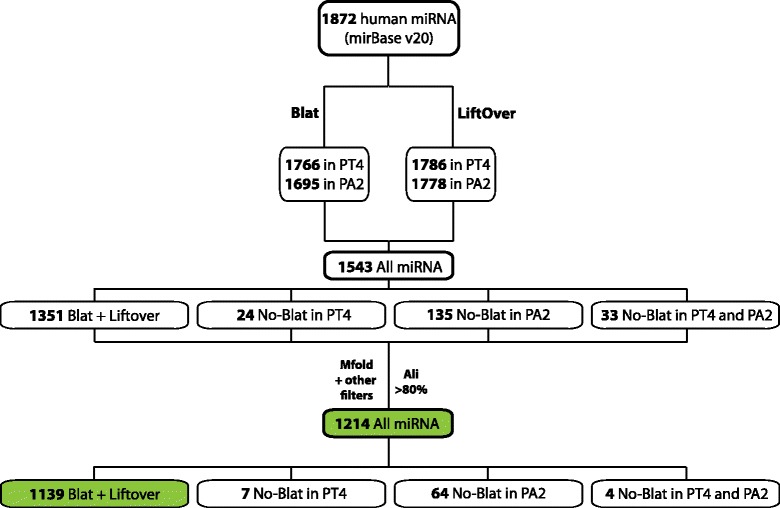
Workflow diagram of miRNA selection and filtering. The two subsets of miRNAs analyzed in this study are indicated in green boxes. Ali = Alignment

To compare the rates of sequence evolution among miRNAs in human and chimpanzee it is necessary to have a reliable set of alignments of the sequences of interest. To locate miRNA orthologs we used the combined information provided by BLAST-like alignment tool (BLAT) and liftOver tools, and we filtered the alignments according to criteria based on sequence identity, proportion of sequence covered, genomic location and secondary structure, thus ensuring that the sequences obtained could be proper orthologs (see Methods). From the 1,872 human miRNAs that were initially considered (miRBase Release 20), 1,766 (94.3 %) and 1,695 (90.6 %) had BLAT hits in the chimpanzee and orang-utan genomes, respectively (Fig. 1). This elevated percentage is in accordance with previous studies [27] and reveals a high conservation of these sequences on these three species. Importantly, miRNAs with no BLAT matches in either one or both species are of particular evolutionary interest as they may be novel human-specific miRNAs or very diverged orthologs. For that reason we kept all miRNAs with or without BLAT matches for additional analyses.

To further refine our analysis and given the importance of a correct secondary structure for miRNA processing, a filtering by minimum free energy (MFE) was applied. This allowed to keep only the putative orthologs with structures whose MFEs were comparable to MFEs of real human miRNAs. In addition, the aligned sequence was requested to span at least 80 % of the length of the human sequence. Nevertheless, given the high rate of duplicated miRNA sequences, a BLAT hit could map to a non-orthologous region on the genomes of chimpanzee and orang-utan. Although these sequences could also be of interest, their evolutionary stories may differ from their paralogs in other *locus*. To solve the possible ambiguities created by highly similar miRNA paralogs, human miRNA coordinates were converted using liftOver and crossed with the coordinates of the BLAT hits previously obtained. We excluded all miRNAs with a “many-to-one” orthologous assignment in either chimpanzee or orang-utan. Out of all miRNAs surviving the filtering process described above, 1,139 (60.8 %) did BLAT and liftOver in both chimpanzee and orang-utan genomes; 64 (3.4 %) did BLAT only in chimpanzee and liftOver only in orang-utan; 7 (0.4 %) did BLAT only in orang-utan and liftOver only in chimpanzee; and, finally, 4 (0.2 %) did only liftOver but not BLAT in both orang-utan and chimpanzee genomes. A final number of 1,214 (64.9 %) alignments constituted the main working set used in this study. However, the more stringent set of 1,139 miRNAs that did BLAT and liftOver in both species was used to test the robustness of some of the results (Fig. 1).

The lack of proper orthologs for a number of miRNAs can be in part the result of gaps in the assemblies of chimpanzee and orang-utan. Also, regions enriched in repetitive sequences, such as ALUs, preclude proper alignment between assemblies and make LiftOver results poorer. A pernicious combination of these factors may have hidden some miRNAs that could be in fact very divergent between species but perhaps of functional and phenotypic relevance. These limitations are difficult to overcome using the current available assemblies but will be partially solved when better assemblies based on high coverage sequencing data become available. We analysed whether our predicted miRNA orthologs in chimpanzee and orang-utan had been already annotated in miRBase (Release 20). We found that 509 predicted filtered orthologs matched coordinates with 509 out of 629 chimpanzee annotated miRNAs (80.9 %). For orang-utan we found that 520 predicted filtered orthologs matched coordinates with 520 out of 632 orang-utan annotated miRNAs (82.3 %). When considering the overlap with the subset of miRNAs before applying quality filters the percentages grow to 91.1 and 91.8 % for chimpanzee and orang-utan, respectively.

### MiRNAs classification

MiRNAs were classified into different categories according to their clustering, number of copies, location with respect to protein-coding genes and phylogenetic distribution (Table 1 and Additional file 1: Table S1). Analysis of the distribution of miRNAs among these categories showed that while multiple-copy miRNAs are equally distributed as either clustered or non-clustered miRNAs, single-copy miRNAs tend to be non-clustered (Fisher *p*-value <0.0001). More than two thirds (840) of all studied miRNAs belong to primate-specific families, in accordance to a previous report by Iwama and colleagues [26] estimating that roughly half of human miRNAs originated within the simian lineage.

**Table 1:** Substitution rates in miRNA categories

							Substitution rates			
1,214 miRNA			miRNAs	Effective Length	Subs. Human	Subs. Chimp	miRNAs Human (sd)	miRNAs Chimp (sd)	Flank. Human (sd)	Flank. Chimpanzee (sd)
840 Primate specific miRNAs	Cluster	Clustered	67	5558	32	39	0.0058 (0.0014)	0.007 (0.0016)	0.0077 (0.0011)	0.0094 (0.0022)
		Non-clustered	773	62048	341	394	0.0055 (0.0003)	0.0063 (0.0005)	0.0065 (0.0003)	0.007 (0.0006)
	Copies	Multiple	73	6323	30	31	0.0047 (0.0009)	0.0049 (0.0008)	0.0092 (0.0013)	0.0075 (0.0015)
		Single	767	61283	343	402	0.0056 (0.0003)	0.0066 (0.0005)	0.0064 (0.0003)	0.0072 (0.0006)
	Localization	Intergenic	200	16539	97	109	0.0059 (0.0007)	0.0066 (0.0008)	0.0082 (0.0008)	0.0093 (0.0013)
		Genic	516	40895	219	242	0.0054 (0.0004)	0.0059 (0.0004)	0.006 (0.0004)	0.006 (0.0006)
		Exon	47	4031	23	19	0.0057 (0.0011)	0.0047 (0.0015)	0.0038 (0.0011)	0.0068 (0.0038)
		intron	371	29243	160	180	0.0055 (0.0004)	0.0062 (0.0006)	0.0065 (0.0006)	0.0063 (0.0006)
374 conserved-beyond-primates mIRNAs	Cluster	Clustered	191	16615	26	40	0.0016 (0.0003)	0.0024 (0.0005)	0.0042 (0.0006)	0.0046 (0.0006)
		Non-clustered	183	16264	21	35	0.0013 (0.0002)	0.0022 (0.0004)	0.0054 (0.0008)	0.0058 (0.0014)
	Copies	Multiple	226	199676	29	44	0.0015 (0.0003)	0.0022 (0.0004)	0.0049 (0.0007)	0.0056 (0.0014)
		Single	148	13203	18	31	0.0014 (0.0003)	0.0023 (0.0005)	0.0048 (0.0006)	0.0045 (0.0007)
	Localization	Intergenic	142	12400	23	42	0.0019 (0.0004)	0.0034 (0.0007)	0.0052 (0.0008)	0.0053 (0.0008)
		Genic	139	12415	15	21	0.0012 (0.0003)	0.0017 (0.0004)	0.0043 (0.0006)	0.0065 (0.0022)
		Exon	7	639	0	0	0 (0)	0 (0)	0.0054 (0.0023)	0.0036 (0.0021)
		Intron	110	9929	12	15	0.0012 (0.0003)	0.0015(0.0004)	0.0042 (0.0006)	0.0072 (0.0021)
Ancestral repeats			1479*100	119996	635.4	712.4	0.0053 (0.0002)	0.0059 (0.0003)	0 (0)	0 (0)
Cluster 14			40	3214	4	6	0.0012 (0.0006)	0.0019 (0.0014)	0.0032 (0.0013)	0.0054 (0.0015)
Cluster 19			28	2417	4	7	0.0017 (0.0007)	0.0029 (0.0011)	0.0103 (0.0021)	0.0085 (0.0026)

Effective length; effectively analysed sequence in base pairs (i.e. Neither gaps nor INDELs); Chimp, chimpanzee; Subs., substitutions in the indicated lineage; sd, standard deviations of substitution rates from 100 bootstraps for miRNAs and flanking regions (Flank.)

We found that the group of primate-specific miRNAs was enriched in single-copy and non-clustered miRNAs (Fisher *p*-value <0.0001 for both) with only 67 miRNAs belonging to miRNA clusters. Because 27 out of these 67 miRNAs belonged to an exceptionally large cluster on chromosome 19 (Cl19); excluding them would reinforce the observation that primate-specific miRNAs are mostly not clustered. On the contrary, miRNAs present in families conserved beyond primates (ConFam miRNAs) are evenly distributed between the clustered and non-clustered categories. We also observed that while ConFam miRNAs were similarly found either in genic or intergenic regions, primate-specific miRNAs were predominantly found in genic regions (Fisher *p*-value <0.0001). This again is in accordance with previous studies reporting that recently emerged miRNAs show higher tendency to be located in genes than more conserved miRNAs [17].

Next, we analysed the distribution in categories of miRNAs whose orthologs had already been described in miRBase for chimpanzee and orang-utan (see above). We found that this subset of annotated miRNAs from both chimpanzee and orang-utan were significantly more depleted of primate-specific, single-copy, non-clustered and intergenic miRNAs (all Fisher’s exact test *P*-values <0.0001) than non-annotated orthologous miRNAs.

### Rates of molecular evolution in individual miRNAs and *in silico* functional analysis

We investigated whether there were miRNAs showing accelerated substitution rates in either human or chimpanzee lineages applying Tajima’s Relative Rate Test (RRT) [35] upon the set of 1,214 miRNA alignments that passed the liftOver and secondary structure filters. Evolutionary rates can be found in Additional file 1: Table S1 (average rate for human being 0.0042 and for chimpanzee 0.0052). More than 50 % of all miRNA alignments (642 miRNAs) were totally identical between humans and chimpanzees, in accordance with the high sequence conservation reported for miRNAs among primates [27]. The largest number of human-specific substitutions found in one miRNA was seven, in hsa-mir-3648, that also contained three substitutions in the chimpanzee lineage. Only four conserved target genes were predicted for this miRNA, which may indicate either little functional relevance or a high degree of specialisation for miR-3648. This miRNA, however, did not reach statistical significance in Tajima’s RRT (Additional file 1: Table S1) and we only had liftOver support to determine its orthology. In fact only three miRNAs surpassed a nominal *p*-value threshold of 0.05 in this test (none passed Bonferroni correction for multiple testing considering a threshold at 0.05/1214 = 0.000041). The first one was hsa-mir-4267 (Tajima’s RRT *p*-value of 0.00007), for which we found 14 mismatches in the chimpanzee lineage; again we only had liftOver support to sustain this miRNA orthology. The other two miRNAs were hsa-mir-4686 and hsa-mir-3691 that contained 12 and six chimpanzee-specific substitutions and presented *p*-values of 0.00031 and 0.02868, respectively.

Next, we predicted target genes for the miRNAs showing the highest substitution rates in each lineage (humans, chimpanzee and orang-utan). We selected the top ranking ten miRNAs with highest relative rate of evolution and obtained their list of predicted target sites in TargetScan, considering conserved and non-conserved target sites. We produced gene enrichment analysis in GO categories of biological processes independently in each species (Additional file 1: Table S2). Categories enriched in each species were different and affected by the inclusion or not of non-conserved target sites. Top significantly enriched categories in targets of human miRNAs with high substitution rates included regulation of signalling, cell communication and signal transduction (GO:0023056, GO:0010647, and GO:0009967), syncytium formation (GO:0000768 and GO:0006949) and astrocytes development and differentiation (GO:0014002 and GO:0048708), among others. The finding of these categories enriched for human accelerated miRNAs is of special interest since an important role for astrocytes in the development of uniquely human traits such as cognition has been suggested [36]. Chimpanzee top enriched categories included RNA processing, transport and localization (e.g. GO:0006364, GO:0051028, GO:0006403 or GO:0051236), among others. Orang-utan top enriched categories included many related to morphogenesis and development of different tissues and organs such as dorsal spinal cord (GO:0021516), limbs (GO:0035108 and GO:0060173) or the camera-type eye development (GO:0043010), and also one category related to adult walking behaviour (GO:0007628), among others.

### Rates of molecular evolution in miRNA categories

Since we had multiple categories and each of them is a variable that may have an effect on miRNA substitution rates, a multiple linear model analysis was performed to reveal the relationship between these variables and the variance in miRNA substitution rates (Additional file 1: Table S3). The primate-specific category was the only that could significantly explain the variance in the linear model including all the studied categories. These results showed that the driving category explaining differences in substitution rates in miRNAs was whether they are primate-specific or not. Once primate-specificity was controlled for, no other category was significantly associated with substitution rates. Naturally, miRNAs belonging to primate-specific category are younger than the rest. It has been postulated that many of the newly emerged lineage-specific miRNAs are but transient forms in the process of being shaped by evolution into functional miRNAs that can be correctly processed by Drosha and Dicer while fitting into regulatory networks in a way that is not deleterious to the organism, in which case they would be removed by natural selection [37].

To test whether the results from applying these linear models could be affected by different alternative filtering criteria we repeated the analysis in different subsets of miRNAs (Additional file 1: Table S3). First we considered a subset that did not include orthologous miRNAs with only evidence from liftOver and no BLAT hits. Second, we considered a subset excluding any many-to-one miRNA, i.e. multiple human miRNA showing best-predicted orthology to the same chimpanzee miRNA. Third, we excluded any miRNA classified as “primate-specific” but showing homology to non-primate sequences in a BLAST search against the nt database. Finally, we also tested a reduced subset of miRNAs in which all predicted chimpanzee orthologs had been annotated in miRBase for chimpanzee. In all cases, the only significant variable was the “primate-specificity” (Additional file 1: Table S3).

Substitution rates in flanking regions were not homogeneous across categories. Flanking regions of primate-specific miRNAs showed significantly less conservation than flanking regions of ConFam miRNAs in many subsets of miRNAs studied (Additional file 1: Table S3). This probably reflects a higher level of background selection acting upon the immediately adjacent sequence of old miRNAs. Also, flanking regions of genic miRNAs are significantly and consistently more conserved than those of intergenic miRNAs (Additional file 1: Table S3), reflecting the effect of purifying selection acting on protein-coding genes.

We conducted a complementary analysis to overcome a possible lack of statistical power due to the small size of miRNA sequences. All miRNA alignments (and, independently, all their flanking regions) were concatenated according to their category (see Methods) and the substitution rates and Tajima’s RRT test were calculated for each category (Table 1 and Additional file 1: Table S4). Tajima’s RRT indicated that, once *p*-values were Bonferroni-corrected for multiple testing, human and chimpanzee miRNA categories evolve at undistinguishable rates (Additional file 1: Table S4).

**Fig. 2:**
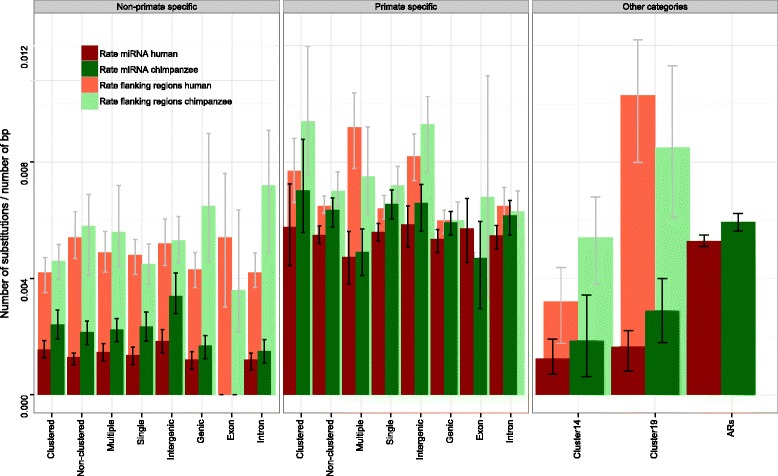
Substitution rates of miRNA categories and neutral references

For each group, a set of one hundred alignments was produced by randomly choosing the same number of miRNAs that compose the category with replacement. These bootstrap datasets were used to obtain confidence intervals for the substitution rate of each category and control for the contribution of outlier miRNAs. The same analysis was performed on ancestral repeats (ARs) and on miRNA flanking regions, also classified by categories (Table 1, Additional file 1: Table S4 and Fig. 2). With the observed values and bootstrap distributions, we compared rates of molecular evolution among different categories only in the human lineage independently in primate-specific and ConFam miRNAs (Fig. 2 and Additional file 1: Table S5). Once miRNA primate-specificity was taken into account, no major differences exist among the remaining categories; which in summary confirmed the similar rates of molecular evolution occurring among the remaining miRNA categories.

Again, in order to discard the possible contribution of dubious orthologous miRNAs (i.e. alignments based only in liftOver) in the results obtained, all previous analyses with the concatenated categories were repeated in the subset of 1,139 miRNAs that did both BLAT and liftOver among species, favouring well conserved miRNA orthologs in exchange for confidence in orthology. We obtained similar results in all analysis (Additional file 1: Table S4 and Additional file 1: Table S5).

Substitutions in the miRNA seed region are likely the most relevant changes affecting miRNA target specificity. Thus, we specifically tested for differences in substitution rates among categories considering the 1,841 seed regions derived form our 1,214 precursor miRNA sequences. We observed that most seeds (97.6 and 97 % in human and chimpanzee, respectively) did not show any lineage specific substitution (Additional file 1: Table S6) with a maximum of two branch-specific substitutions occurring in one miRNA, hsa-mir-1178, in human and in two miRNAs, hsa-mir-1200 and hsa-mir-4507, in chimpanzee. The multiple regression model between substitution rates in seed regions and categories replicated the results obtained for the precursor sequences: the only significant explanatory variable of the model was the primate-specificity category and primate-specific miRNAs displayed significantly higher substitution rates in seed regions than ConFam miRNAs. This observation was also robust to alternative filtering criteria of orthologs (Additional file 1: Table S3).

The finding of different selective pressures acting on young and old miRNA genes is in agreement with the results obtained by Meunier [21] using a different approach in which they compare nucleotide substitution rates in human miRNA with the genomic background across primates using a phylogenetic method (phyloP [38];). Through our approach we have confirmed these observations while controlling for possible confounders such as different miRNA categories or non-adaptive forces, such as rapid evolution at CpG dinucleotides and/or the contribution of GC-biased gene conversion (BGC) and differences in nucleotide composition. To gain insight into the real cause for enhanced substitution rates in primate-specific miRNAs, we compared the proportion of C to T transitions in CpG dinucleotides between primate-specific and ConFam miRNAs and did not find statistically significant differences between both groups, thus indicating that CpG hyper-mutability did not explain the observed differences in substitution rates (Additional file 1: Table S7). BGC is a process that favours fixation of strong (GC bond)-to-weak (AT bond) (S > W) mutations as a consequence of molecular events related to recombination. Regions of the genome close to old recombination hotspots can thus present increased divergence and be missinterpreted as subjects of positive selection. We found that ConFam miRNAs showed a significantly lower W > S bias compared to primate specific miRNA (Additional file 1: Table S8), the latter showing a W > S bias of around 0.39, consistent with the genome-wide substitution pattern previously found using human-chimpanzee-macaque alignments [39]. The W > S bias also extended to miRNA flanking regions, indicating that the effect is noticeable in regions larger than the few base pairs (bp) analysed, as previously described in exons [39]. The fact that ConFam miRNAs showed a lower fixation rate of mutations driven by BGC further supports our observation that this category of miRNAs is subjected to a stronger purifying selection than primate-specific miRNAs. Excluding W > S changes, we also find that primate-specific miRNAs at least doubled substitution rates in all other type of changes (S > W, S > S + W > W and S > W+ S > S + W > W) (Additional file 1: Table S8). Differences in nucleotide composition besides CpG and biased gene conversion could also contribute to explain differences in substitution rates, since different nucleotides show different propensity to become a substitution [40]. We calculated substitution rates in the human branch for each type of nucleotide substitution and found that for all types primate-specific miRNA at least doubled ConFam substitution rates (Additional file 1: Table S9).

### Maximum Likelihood Testing for accelerated evolution in miRNAs

In addition to our previous analysis a Maximum Likelihood Testing test was implemented in HyPhy [41] to search for accelerated evolution in miRNA sequences at the level of both, individual miRNAs and category-based concatenated groups. First, individual miRNA sequences were compared to a set of random ARs. No miRNA resisted multiple-testing correction, which convinced us that the short length of individual miRNAs precludes a properly powered analysis for positive selection at this level. As for concatenated miRNA groups, they were compared to random concatenated ARs as well as to their concatenated flanking regions, both of similar sequence length than miRNAs. No miRNA category showed significant acceleration compared to either ARs or miRNA flanking regions (Additional file 1: Table S10).

ARs and flanking regions present particular advantages as neutral references. While ARs are widely considered to evolve neutrally, they might be located far from the analysed miRNA. Since different parts of the genomes can be subjected to different rates of divergence, the use of surrounding flanking regions could be advisable in this sense. In exchange for that advantage, flanking regions can be subjected to linked selection of the surrounding areas, might they be protein-coding genes, other non-coding genes (as noted in [34]) or even the miRNAs themselves. After performing the corresponding comparisons we obtained similar results with both neutral references, confirming the constriction of the miRNAs categories analysed.

### Substitution rates in miRNA clusters from chromosomes 14 and 19

Most human miRNA clusters are composed of 2 to 8 members, and it has been shown that 68.49 % of them involve only two miRNAs [15]. But there are two notable exceptions: a cluster specific of placental mammals carrying 42 miRNAs in chromosome 14 (Cl14) and another primate-specific cluster on chromosome 19 harbouring 46 miRNAs (Cl19). Due to the evolutionary relevance reported for these two clusters [42–44] we aimed to analyze their rates of molecular evolution as separate entities.

For Cl14, 40 out of 42 (95.2 %) miRNAs could be analyzed, while for Cl19, only 28 out of 46 miRNAs (60.9 %) could be analyzed (Table 1 and Additional file 1: Table S11). Most of the exclusions from Cl19 were due to discrepancies between BLAT and liftOver (some of them mapped to chr19_random in the ponAbe2 orang-utan genome assembly). This is indicative of a higher sequence complexity in the Cl19 region compared to Cl14. Actually, Cl19 emerged from a primate-specific expansion of miRNA driven by ALUs, which have been found enriched in this region of the chromosome 19 [45]. Cl14, in contrast, is rich in ConFam miRNAs [42] and its genomic region appears depleted of ALUs [46].

Three-way alignments and flanking regions from Cl14 and Cl19 were concatenated independently and their aggregated substitution rates were calculated. No significant differences were observed between both clusters. Surprisingly, the 28 miRNAs analysed for Cl19, although being primate-specific, showed levels of constraint similar to those of any other category depleted of primate-specific miRNAs. The only remarkable difference in substitution rates was observed between the clustered miRNAs and their flanking regions (Fig. 2), being those for Cl19 much higher than in Cl14 and than in any other category, including ARs. This may reflect the increased divergence around non-allelic homologous recombination breakpoints occurred in this ALU-enriched region [45]. In order to test the effect of surrounding ALUs in the divergence of miRNAs and flanking regions, we identified all ALUs present in flanking regions and beyond (within regions of 100 bp or 500 bp at both sides, see Additional file 1: Table S12). Clearly, chromosome 19 showed the higher number of surrounding ALUs, corrected by the number of miRNAs present in the chromosome. However, diversity in flanking regions was not affected by ALUs.

## Conclusions

In this study we created a carefully curated set of sequences that are putative orthologs to human miRNAs in chimpanzee and orang-utan. We estimated their substitution rates both independently and in categories and compared with two sets of sequences as neutral references, ARs and flanking regions. We found that although more than 50 % of human miRNAs are perfectly conserved among these three species, the category of novel primate-specific miRNAs is accelerated in comparison to miRNAs that are also present in other lineages. Our results corroborate the remarkable conservation noted for these regulators, which is a proxy of their functional relevance, but also points that a subset of miRNAs may evolve differently reflecting the effect of weaker purifying selection on these genomic regions or even potentially being under the action positive selection. It is plausible that many of these newly emerged miRNAs are not yet established miRNAs but miRNAs in transient phases, from which some may be maintained on the genomes while others, may be lost.

One of the limitations of this study is the use of putative orthologous miRNAs predicted from extrapolation of human miRNAs in other genomes by sequence conservation and MFE compatible with a miRNA hairpin, which does not necessarily imply that these orthologous miRNAs effectively exist in other species. On the other hand existing orthologs in more distant species have probably accumulated too many substitutions to be identified. Firstly, even if the sequence of the hairpin is identical, differences on their cis-regulatory region could make them inactive on the compared species. Secondly the few changes observed may impair their proper processing and nullify their action. The small size and high number of paralogous copies of miRNAs are inherent problems for cross-species comparisons. Many miRNAs have been claimed to be human-specific when in fact they were not detected on other genomes due to low quality assemblies. Bioinformatic comparisons of miRNA complements will be greatly boosted as new assemblies for more species are developed. To uncover whether a putative miRNA ortholog is active in other species we would need the generation of more expression and functional data. Lately, some deep RNA sequencing studies have advanced our knowledge of the miRNAs present in different tissues of non-human primates such as chimpanzee, gorilla, orang-utan and rhesus macaque [29], although no particular attention has been paid to the existence of species-specific miRNAs. This is probably the reason for which the number of miRNAs described in human still more than doubled the numbers in closely related species. Deciphering the real repertoire of miRNAs present in other primate species through a combination of genome sequencing, gene expression analysis in a wide battery of tissues and functional studies would help unravelling the role of miRNAs in primate evolution.

## Methods

### Obtaining alignments of orthologous miRNAs

Genome coordinates in the hg19 genome assembly from the 1,872 known human miRNA precursors were retrieved from miRBase release 20 ([30], www.mirbase.org). In order to obtain good quality alignments of miRNA orthologs in chimpanzee and orang-utan we followed a number of steps summarized in Fig. 1. First, we performed a BLAT search [47] of each miRNA precursor reported in human against chimpanzee and orang-utan assemblies (panTro4 and ponAbe2, respectively). A given miRNA could either i) present a perfect or imperfect full match with one or multiple hits on the other assemblies; ii) show a perfect or imperfect partial match, once or multiple times; or iii) show no matches on the other assemblies. BLAT matches of less than 80 % of the query length were classified together with non-matching miRNAs. To obtain the best hit for miRNAs matching multiple times, we consecutively ordered matches by length and identity and retrieved the best-ranked ones. To solve ambiguous multiple matches and to reassure partial or imperfect matches we performed liftOver of human miRNA coordinates on chimpanzee and orang-utan assemblies. For each miRNA, we intersected liftOver and BLAT coordinates and obtained the leftmost and rightmost positions of the intersecting set of coordinates. To avoid biases against most divergent miRNAs with either no BLAT matches or <80 % in length hits, we kept three categories of miRNAs that included them: i) miRNAs with only BLAT hits in chimpanzee but not in orang-utan, ii) miRNAs with only BLAT hits in orang-utan but not in chimpanzee, and iii) miRNAs with no BLAT hits in neither chimpanzee nor orang-utan. In these three cases, coordinates with no hits in BLAT were taken only from the liftOver. We excluded 88 miRNA that were assigned multiple times to the same loci in chimpanzee and orang-utan assemblies, indicating a many-to-one human-primate orthology considering assemblies used.

We then extracted genomic DNA sequences from all the coordinates of putative miRNAs orthologs. Finally, for each miRNA we re-aligned the three extracted sequences with ClustalW-2.1 [48] and trimmed the extremes of the resulting alignment to include only the positions spanned by the human miRNA sequence. Taking a conservative approach, only multiple sequence alignments covering at least 80 % of human miRNA sequence were considered for further analysis. We also filtered-out alignments with internal gaps in the hg19 sequence larger than 20 nucleotides.

To obtain alignments of each precursor miRNA seed region we first obtained absolute coordinates of seed regions in the human assembly from miRBase. We then calculated and extracted the relative positions of seed regions within each miRNA alignment.

### Filtering of miRNAs based on folding minimum free energy

Even when human miRNAs show BLAT hits in coincidence with liftOver coordinates, it is still possible that the deduced orthologous sequences are not compatible with a miRNA secondary structure. To identify and exclude from analysis such false miRNA orthologs, we calculated the miRNA folding MFE for all known human miRNAs using Mfold [49] and compared them with the folding free energies of our putative orthologs. We filtered out miRNA three-way alignments in which any of the three orthologous sequences did not show a MFE comparable with those of true miRNAs hairpins (average MFE of -37.9), i.e. showed MFE values beyond the 5 % right-tail of the MFE distribution for all human miRNAs.

### Extraction of sequences from miRNA flanking regions

For all miRNA alignments we obtained the multiple sequence alignments of their 5’ and 3’ flanking regions. We targeted 40 bp at each flank to end up with approximately 80 bp of total flanking region, which is close to the average length of human miRNA sequences in miRBase v20 (80.7 bp). To do so, we extracted 100 bp from the start to end coordinates of the previous BLAT and liftOver analyses. We corrected sequences to keep the strand coherence when needed. We repeated the multiple sequence alignment with ClustalW-2.1, trimmed and kept the 40 bp immediately before and after the human miRNA sequence. We filtered out miRNAs that showed strand discrepancies between the alignments of the miRNA sequence and its flanking region. We also did not considered miRNAs with aligned flanking regions spanning less than 50 % of the targeted 80 bp.

### Ancestral repeats

A full coordinate set of ARs in hg18 assembly was kindly provided by Professor Webb Miller. We used liftOver to obtain coordinates for ARs in hg19, panTro4 and ponAbe2 and kept only those ARs that survived reciprocal liftOver in all three assemblies. We also filtered out those ARs that were either shorter than 70 bp (the first quartile of all human miRNAs length distribution) or longer than 91 bp (the third quartile). To ensure proper orthology we only retained ARs that make liftOver to the same chromosome. We obtained the sequences from the assemblies, and finally we aligned them while correcting coherence in strand orientation using PAGAN.

### miRNAs classification into categories

We classified all miRNAs described in humans and their putative orthologs according to four different criteria. First, miRNAs were divided as either *clustered* or *isolated*; a cluster was defined as two or more miRNAs located at less than 10 Kb from the next one in the same strand. The second criterion took into account the number of copies of a given miRNA in the genome; we used the miRNA families from miRBase to classify miRNAs as either *multiple-copy*, when the miRNA pertained to a family with two or more members, or *single-copy* in all other cases. A third classification took into account the phylogenetic distribution of miRNAs as follows: the miFam.dat file containing all reported miRNAs grouped into families was downloaded from miRBase release 20. Three kinds of families were identified: those containing only miRNAs reported in primates (primate-specific), families containing miRNAs in primates and in other species (they are families conserved beyond primates, which we abbreviate as ConFam) and families for which none of the miRNAs were reported in any primate species (no-primate). There were also miRNAs in human that were not reported in any other species, these orphan human miRNAs were also regarded as primate-specific. The fourth category classified miRNAs as either intergenic, when they were located at more than 50 Kb from a protein-coding gene, or genic, when the miRNA was found in an exon (if one or more nucleotides overlapped with an annotated exon), intron or UTR of a gene. For the analyses at the level of categories, miRNA alignments were concatenated in a single one for each category using a Perl script.

### Counting nucleotide substitutions and Tajima’s RRT

The number of substitutions occurred in human and chimpanzee branches was obtained by orienting each position in ancestral and derived states using the orang-utan allele. Then, for each individual miRNA or miRNA category, we calculated the substitution rate by dividing the observed number of branch-specific nucleotide substitutions over the total aligned miRNA length, insertion-deletion (INDEL) positions were not counted in the total number of sites. To compare the rates of molecular evolution between human and chimpanzee, we performed Tajima’s RRT [35] for each miRNA and miRNA category, the latter obtained by concatenating miRNAs. We considered only branch specific substitutions that could be oriented with orang-utan (i.e. ignoring multiallelic positions). Confidence intervals for substitution rates in each category of miRNAs were achieved by bootstrapping miRNAs within each category 100 times.

### CpG sites and GC-biased gene conversion

To analyze the contribution of CpG sites to substitution rates, we counted the human-specific, chimpanzee-specific and aggregated substitutions consisting in CpG to TpG transitions (also considering the reverse strand, i.e. CpG to CpA). We required nucleotide G in CpG sites and C in CpA sites to be conserved between human and chimpanzee. We compared the proportion of CpG substitutions to non-CpG substitutions between miRNA categories using a Fisher’s exact test. We assessed the contribution of GC-biased gene conversion to miRNA substitution rates counting all strong-to-weak (*S > W*) nucleotide changes (i.e. C > A, C > T, G > A, G > T) and weak-to-strong (*W > S*) changes (i.e. A > C, A > G, T > C and T > G) occurring between human and chimpanzee or specifically in each lineage and then compared the proportion of *S > W* to *W > S* substitutions between categories using a Fisher’s exact test. W > S bias was calculated as: W > S bias = n_w>S_/(n_w>S_ + n_S>W_).

### Accelerated rates of evolution using HyPHY

We used HyPHY [41] to test for accelerated evolution in miRNAs as Haygood and colleagues did in their study in gene promoters [50]. To do so we used both ARs and miRNA flanking regions as neutral reference sequences. For each miRNA or miRNA category we ran the test against 100 random neutral reference sequences: 100 random ARs or 100 random miRNA flanking regions of the same category tested with replacement. Each of the 100 tests was run 100 times and the best likelihood was obtained. We obtained the median *p*-value from these sets of 100 runs.

### Statistical analysis

The general linear model and other statistical analysis were performed using R. The set of variables tested in each model and the total number of miRNAs involved in each one are shown in Additional file 1: Table S3. Differences in substitution rates between categories in concatenated alignments were determined after calculation of the represented percentile in the distribution of 100 randomizations of the compared category. Category values were considered significantly different when the observed values fall reciprocally in the tails of the distribution, considering percentiles 2.5 and 97.5 % as thresholds.

### Gene enrichment analysis

Gene enrichment analysis was performed using R packages topGO based in Alexa [51] and goseq [52] which tests for enrichment in “Biological Process” functional categories in *non-slimmed* Gene Ontology (GO). Since hyper geometric test alone can not account for underlying biases in the prediction of miRNA targets [53], we performed 1000 randomizations of groups of 10 miRNAs (when testing the top 10 accelerated miRNAs) or 1000 randomizations of a matched number of targets (when testing individual miRNAs) and recalculated the classic Fisher test. We then required that the empirical *p*-value for the same category obtained was greater than the observed *p*-value in more than 990 permutations (i.e. *p*-value < 0.01). All the genes represented in TargetScan 6.2 ([54], HYPERLINK “http://www.targetscan.org/”) that were predicted as target for at least one miRNA of our dataset were used as background to test the enrichment of our sets of predicted targets. Since targets might not be perfectly conserved particularly in orthologous miRNAs in chimpanzee and orang-utan, we also considered non-conserved predicted targets from TargetScan and required a total context score of ≤ -0.4 before performing the enrichment as in Jansen [55].

## Abbreviations

ARs, ancestral repeats; BGC, GC-biased gene conversion; BLAT, BLAST-like alignment tool; bp, base pairs; Cl14, microRNA cluster on chromosome 14; Cl19, microRNA cluster on chromosome 19; ConFam miRNAs, miRNAs present in families conserved beyond primates; flank, Flanking regions; GO, Gene Ontology; INDEL, insertion-deletion; MFE, minimum free energy; miRNA, microRNA; nt, nucleotide; RISC, RNA-Induced Silencing Complex; RRT, Relative Rate Test; S, strong (GC bond); sd, standard deviations; snoRNAs, small nucleolar RNAs; ts, transitions; tv, transversions; W, weak (AT bond)

## Additional files

Additional file 1: Table S1. Statistics for miRNA tested in Tajima’s Relative Rate Test and HyPHY analyses. Type, Mapping result in PT4 (100 means perfect match). L.hg19, length of miRNAs in humans. True_length, Length trully informative. Median *p*-value indicates the median of 100 *p*-values obtained from HyPHY analysis. pVal.Tajimas indicates the *p*-value of the Tajima’s RRT. Qvalue, q-value calculation of the previous distribution of *p*-values. Type indicates whether miRNA map using BLAT in all all species (TOTAL), only in chimpanzee (NOMAP_PA2) or only in orang-utan (NOMAP_PT4). Mut, number of substitutions found in each branch. Categories: Number of copies (M), primate-specificity (P), localization in genome (L1), localization within genes (L2) and clustering (C). **Table S2.** Functional enrichment analysis of TargetScan predicted target genes for miRNAs with significant results on Tajima’s test. The classic Fisher’s test and 1000 randomizations of groups of ten miRNAs was used for the analysis of hsa-mir-3691, hsa-mir-4267 and hsa-mir-4686. **Table S3.** Linear models tested and their *p*-values. The number of miRNAs tested in each model is indicated in bold. B stands for BLAST and L for Liftover. Final subset: The subset of miRNAs described in the manuscript. BLAST filtered: Primate-specific miRNAs showing BLAST hits in other non-primate species are removed. Only in miRBase miRNAs whose predicted chimpanzee orthologs are also described in miRBase for chimpanzees. With many-to-one: A subset containing miRNAs presenting multiple human versions assigned to the same chimpanzee or orang-utan ortholog. Categories: Number of copies (M), primate-specificity (P), localization in genome (L1), localization within genes (L2) and clustering (C). **Table S4.** Statistical test for miRNA categories and results for the Tajima’s test. Effective length indicates sequence (number of base pairs) effectively analysed (i.e. No gaps nor INDELs). First pval column contains the *p*-value for Tajima’s test for Human-Chimpanzee comparison. The second pval column indicates the *p*-value for Tajima’s test using information on transitions (ts) and transversions (tv), contained in the previous four fields. subs, substitutions in the indicated lineage; rate, substitution rate in the indicated lineage. Sd columns contain the standard deviations of substitution rates from 100 bootstraps from miRNAa and flanking regions. **Table S5.** Statistical test of substitution rates of miRNA categories. Green cells indicate significant comparisons at 0.025 tails (see Methods). Two groups of miRNAs are analysed. 1) 1,241 all, miRNAs. 2) 1,139, only miRNAs doing BLAT and Liftover. The number in cells indicates the percentile that any observed value for categories, in rows, occupies in the distribution of 100 bootstraps values for any other category, in columns. **Table S6.** Substitutions found in seed regions in all miRNA analysed. Categories: Number of copies (M), primate-specificity (P), localization in genome (L1), localization within genes (L2) and clustering (C). Subs, substitutions in the indicated lineage. **Table S7.** Analysis of the proportion of C to T transitions in CpG dinucleotides in each miRNA category. Subs, substitutions in the indicated lineage. Prop. CpG, proportion of substitutions in CpG sites. **Table S8.** Analysis of the contribution of GC-BGC in each miRNA category. *P* = PValue, W = Weak, S = Strong. **Table S9.** Rates of substitutions observed for each type of nucleotide in the human lineage. **Table S10**. Median *p*-values for 100 repetitions of the Hyphy analysis for different categories of miRNAs. Either ARs or flanking regions were used as neutral reference sequence. **Table S11**. miRNAs present in clusters of chromosomes 14 and 19. In yellow miRNAs that have been excluded from the study and the reason to do so. **Table S12**. Presence of ALUs near-by miRNAs and diversity in miRNAs and flanking regions for each chromosome. *P*-values and correlation coefficient for divergence measures versus number of ALUs (below). (XLSX 408 kb)
